# Developmental Changes of the Ovary in Neonatal Cotton Rat (*Sigmodon hispidu*s)

**DOI:** 10.3389/fphys.2020.601927

**Published:** 2021-01-13

**Authors:** Md. Rashedul Islam, Osamu Ichii, Teppei Nakamura, Takao Irie, Md. Abdul Masum, Yuki Otani, Takashi Namba, Tsolmon Chuluunbaatar, Yaser Hosny Ali Elewa, Yasuhiro Kon

**Affiliations:** ^1^Laboratory of Anatomy, Department of Basic Veterinary Sciences, Faculty of Veterinary Medicine, Hokkaido University, Sapporo, Japan; ^2^Department of Surgery and Theriogenology, Faculty of Animal Science and Veterinary Medicine, Sher-e-Bangla Agricultural University, Dhaka, Bangladesh; ^3^Laboratory of Agrobiomedical Science, Faculty of Agriculture, Hokkaido University, Sapporo, Japan; ^4^Section of Biological Safety Research, Chitose Laboratory, Japan Food Research Laboratories, Chitose, Japan; ^5^Laboratory of Veterinary Parasitology, Faculty of Agriculture, University of Miyazaki, Miyazaki, Japan; ^6^Department of Anatomy, Histology and Physiology, Faculty of Animal Science and Veterinary Medicine, Sher-e-Bangla Agricultural University, Dhaka, Bangladesh; ^7^Department of Basic Science of Veterinary Medicine, School of Veterinary Medicine, Mongolian University of Life Science, Ulaanbaatar, Mongolia; ^8^Department of Histology, Faculty of Veterinary Medicine, Zagazig University, Zagazig, Egypt

**Keywords:** neonatal cotton rat, folliculogenesis, multi-oocyte follicles, double nucleated oocytes, apoptosis, oocytogenesis

## Abstract

The reproductive characteristics and ovarian development in cotton rats (*Sigmodon hispidus*, CRs) are unclear, although CRs are commonly used as animal models in biomedical research. We previously reported that young (6–8 weeks) CRs showed multi-oocyte follicles (MOFs) and double nucleated oocytes (DNOs) in different stages of follicles. The developmental changes in neonatal CR ovaries were investigated in the present study and were compared with our findings in previous studies of unique phenotypes, particularly in oocytes. CR ovaries at postnatal days (PND) 0, 4, and 7 were obtained from the Hokkaido Institute of Public Health. Samples were analyzed by light and transmission electron microscopy. The general histology and folliculogenesis in CR ovaries were similar to those in other experimental rodents. However, DNOs were observed in all age categories and were frequently observed in primordial follicles, whereas MOFs started to develop from PND4 with greater frequency in primary follicles. Almost all developing follicles expressed DEAD (Asp-Glu-Ala-Asp) box polypeptide 4 and forkhead box L2, which are representative markers of oocytes and follicular epithelial cells, respectively. Ki-67 staining demonstrated the proliferative activity of granulosa cells, but not of oocytes, in follicles. Moreover, rapid folliculogenesis of CR due to a small number of apoptotic oocytes was suggested, based on results of the terminal deoxynucleotidyl transferase dUTP nick end labeling assay, confirming the formation of DNOs or MOFs. These findings clarify the development of unique phenotypes of neonatal CR ovaries and support it as a useful model to better understand folliculogenesis and oocytogenesis as well as their abnormalities in humans and other animals.

## Introduction

Fertility and the entire reproductive lifespan of females depend on the limited and non-restorable reserves of oocytes in neonatal ovaries. Follicle assembly and development have been well documented in many mammalian species (Byskov and Lintern-Moore, 1973; Pepling and Spradling, 2001; Sawyer et al., 2002; Tingen et al., 2009). However, processes that occur at mid-gestation in humans (Hirshfield, 1991; Maheshwari and Fowler, 2008) and during the first postnatal days (PNDs) in rodents remain unclear (Pepling, 2012; Tingen et al., 2009). The germ cell nest is an important developmental stage in the formation of the germline. The nest is evolutionarily conserved in males and females of species ranging from higher insects to frogs, rodents, and other vertebrates (Pepling et al., 1999). In each of these organisms, most of the single primordial germ cells divide synchronously with incomplete cytokinesis or nuclear divisions to form a cluster of cells connected by intercellular bridges (Gondos et al., 1971; Pepling and Spradling, 1998). Nest breakdown involves the degeneration of many germ cell nuclei and the invasion of the pregranulosa cells into the germ cell nests (Pepling and Spradling, 2001). After breakage, the majority of ovarian follicles enclose only a single oocyte, although reports have described two or more oocytes within a single follicle in the ovaries of several mammalian species (Hartman, 1926; Gondos et al., 1971; Lucci et al., 1999; Smitz and Cortvrindt, 2002; Balciuniene et al., 2006; Stankiewicz et al., 2009; Payan-Carreira and Pires, 2008; Yang and Fortune, 2008; Silva-Santos et al., 2013). Furthermore, single oocytes or multi-oocytes are surrounded by a single layer of flattened squamous or multi-layer cuboidal somatic cells termed granulosa cells (GCs). The theca cell layer starts to develop from secondary follicles (SFs). Less attention has been paid to the role of theca cells in follicular function compared to that of GCs (Tajima et al., 2007). However, the communication between oocytes and GCs along with theca cell layers leads to the survival of normal follicles, and its disturbances.

The reproductive characteristics and ovarian development in cotton rats (*Sigmodon hispidus*, CR) remain unclear, even though CRs have been well characterized and are commonly used in biomedical research (Faith et al., 1997). Hispid CRs, like other rodents, can potentially breed year-round, and a single adult female typically produces three to four litters annually, averaging five to seven young per litter. Geographic variation in litter size depends on the ovulation rate, which is influenced by the number of ovulating ovaries. Copulation also increases ovulation rate in some populations (Oswald and Mcclure, 1985). CRs can be reproductively active for 30–40 days. We found that female CRs have several unique phenotypes associated with the urogenital system, such as chronic kidney disease and pyometra (Ichii et al., 2018). Female CRs also display significantly more features of severe chronic kidney disease than males, especially inflammatory cell infiltration and dilation of distal tubules (Ichii et al., 2016). We also documented unique phenotypes in the CR ovary at 6–8 weeks of age, such as multi-oocyte follicles (MOFs) and double nucleated oocytes (DNOs) (Islam et al., 2020). Based on the results, we considered the mechanism of development of MOFs or DNOs as a correlation between oocytes with incomplete cytoplasmic or nuclear division and GC expansion at 6–8 weeks of age. However, their appearance in perinatal CRs remains unclear.

In the present study, we examined the mechanisms of development and compared the characteristics of unique phenotypes in neonatal CRs at PND0 to PND7 with our previous findings using histological analysis. The data increase the understanding of folliculogenesis and oocytogenesis in other mammals, including humans, by considering the appearance of MOFs and DNOs in the CR ovary.

## Materials and Methods

### Animals and Tissue Processing

Animal experiments were performed according to the guidelines of the Hokkaido Institute of Public Health (Sapporo, Japan; approval no. K27-03) and the Faculty of Veterinary Medicine, Hokkaido University (approval no. 20-0012). CRs were maintained as the HIS/Hiph strain through continuous inbreeding under conventional conditions at the Hokkaido Institute of Public Health. Inbred C57BL/6N and outbred Jcl:ICR mice were obtained from Japan SLC Inc. (Hamamatsu, Japan). All CRs and mice were maintained under specific pathogen-free conditions. Food and water were provided *ad libitum*. Twenty-four CRs (eight in each age group), 15 C57BL/6N (five in each age group), and 15 ICRs (five in each age group), and their ovaries were collected at PND0, PND4, and PND7. Each tissue was fixed in 10% neutral buffer formalin (NBF), 4% paraformaldehyde (PFA), or 2.5% glutaraldehyde (GTA) in 0.1 M phosphate buffer (PB) for histological analysis, immunohistochemistry, terminal deoxynucleotidyl transferase dUTP nick end labeling (TUNEL) assays, and ultrastructural analysis. We used five ovaries from each age group for histological analysis. The remainder were used for immunofluorescence, immunohistochemistry, TUNEL assay, and ultrastructural analysis.

### Histological Analysis

Paraffin-embedded ovaries from each age group (five ovaries) of CRs and C57BL/6N mice were cut to a thickness of 2 μm to obtain whole ovarian sections. The sections were stained with hematoxylin-eosin (HE) or periodic acid Schiff-hematoxylin (PAS-H) to examine ovarian morphology in neonatal CRs and mice. The stained sections mounted on glass slides were scanned using a NanoZoomer 2.0 RS virtual slide scanner (Hamamatsu Photonics, Shizuoka, Japan). The data were used for histomorphometry. Microscopy examination was done using a model BZ-X710 microscope (Keyence, Osaka, Japan) to obtain the histological images.

For histomorphometry using HE-stained sections, ovarian follicles were classified, either in nest as cluster form or based on their stages, as primordial (PrF), primary (PF), secondary (SF), and tertiary follicles (TF), according to previously reported studies in rabbits (Al-Mufti et al., 1988) and CRs (Islam et al., 2020). Their numbers were counted every five sections. The number of various developing follicles was multiplied by five to determine the total number of follicles per ovary (Su et al., 2013; Gaytán et al., 2014; Islam et al., 2020). Only follicles containing visible oocytes were counted to avoid double counting. The number and percentages of developing follicles were counted from the total number of follicles, excluding nests. PrFs had an oocyte surrounded by a single layer of flattened follicular epithelial cells (FECs). PFs were identified as oocytes enclosed by a layer of GCs. SFs contained two or three layers of GCs surrounding the oocyte, but there was no visible antral cavity among FECs. TFs with small follicular spaces between FECs began to form, and these follicles were composed of four or more GC layers.

The developmental stage of ovarian follicles containing more than two oocytes (defined as MOFs) or two nuclei (defined as DNOs) were classified according to our previous study (Islam et al., 2020). In neonates, DNOs appeared from PND0, and MOFs from PND4. They were enumerated. The percentage of MOFs or ovarian follicles containing DNOs among all follicles was also calculated.

### Immunofluorescence

The oocyte marker DEAD (Asp-Glu-Ala-Asp) box polypeptide 4 (DDX4) and the FEC marker fork head box L2 (FOXL2) were analyzed to determine whether the oocytes of CRs, in particular those in MOFs or DNOs, showed biological characteristics similar to those of other rodents. Briefly, DDX4 and FOXL2 were expressed in germ cells and FECs from the time of their first appearance to adulthood in mice (Yamashita et al., 2015). Deparaffinized sections were treated with 10 mM citrate buffer for 20 min at 105°C for antigen retrieval. These sections were then blocked with 5% normal donkey serum (Sigma-Aldrich, St. Louis, MO, United States). They were incubated overnight with rabbit anti-DDX4 (1:500 dilution; ab13840; Abcam, Cambridge, United Kingdom) and goat anti-rabbit FOXL2 antibody (1:1000; ab5096; Abcam) at 4°C. The sections were incubated for 1 h at room temperature with Alexa Fluor-488 labeled donkey anti-rabbit IgG (1:500, Life Technologies, Carlsbad, CA, United States) and Alexa Fluor-564 labeled donkey anti-goat IgG (1:500, Life Technologies) for DDX4 and FOXL2 double immunofluorescence. The sections were counterstained with Hoechst 33342 (1:200; Dojindo, Kumamoto, Japan). The stained sections were examined using a BZ-X710 microscope (Keyence).

### Immunohistochemistry

For immunohistochemical staining of Ki-67, deparaffinized sections were treated with 10 mM citrate buffer for 20 min at 105°C for antigen retrieval, and incubated in 0.3% hydrogen peroxide (H_2_O_2_)/methanol solution for 10 min to quench endogenous peroxidase activity. The sections were then blocked with 10% normal goat serum using the SABPO kit (Nichirei Bioscience, Tokyo, Japan) for Ki-67 staining. Sections were incubated overnight with rabbit anti-Ki-67 antibody (1:800; ab15580; Abcam) at 4°C. This antibody has been reported to show positive immunoreactivity in rat GCs (Urata et al., 2020). Negative controls were run with normal rabbit IgG (sc-2027; Santa Cruz Biotechnology, CA, United States). The sections were then treated with biotinylated goat anti-rabbit IgG using the SABPO kit (Nichirei Bioscience) for 30 min at room temperature. This was followed by incubation with streptavidin-horseradish peroxidase using the SABPO kit (Nichirei Bioscience) for 30 min, followed by incubation with 3,3-diaminobenzidine tetrahydrochloride in H_2_O_2_. Finally, the sections were counterstained with hematoxylin and dehydrated using ascending grades of alcohol. The stained sections were examined using a BZ-X710 microscope (Keyence).

### TUNEL Assay

Apoptosis was assessed by TUNEL assay using the *In Situ* Apoptosis Detection Kit (ab206386; Abcam). Collected neonatal ovaries (five in each age group) were fixed in 4% PFA, embedded in paraffin, and sectioned to a thickness of 3 μm. The five largest CR and ICR cross sections (15 μm interval) were placed in a slide from five ovaries of each age group at PND0, PND4, and PND7. The staining procedure was performed according to the manufacturer’s instructions. Briefly, after deparaffinization, the sections were incubated with proteinase K at room temperature for 20 min. Endogenous peroxidase was inactivated by 3% H_2_O_2_/methanol for 5 min. The slides were incubated with TUNEL reaction mixture in a humidified chamber at 37°C for 90 min, followed by washing with PBS. The TUNEL reaction mixture consisted of an enzyme solution (terminal deoxynucleotidyl transferase) and nucleotide mixture. The slides were incubated with a converter anti-fluorescein antibody conjugated with anti-fluorescein isothiocyanate (FITC) horseradish peroxidase (HRP) at 37°C for 30 min. After washing with PBS, the immunoreaction was visualized by incubating with 3,3-diaminobenzidine tetrahydrochloride in H_2_O_2_. Sections were counterstained with methylene green and incubated for 1–3 min. The stained-glass slides were scanned using a NanoZoomer 2.0 RS virtual slide scanner (Hamamatsu Photonics). A model BZ-X710 microscope (Keyence) was used to obtain the images. For TUNEL histomorphometry, the number of positively stained oocytes was counted and averaged using five sections of different stages of each ovary of CRs and ICR mice.

### Electron Microscopy

Cotton rats ovaries (three ovaries from each age group) were collected and immediately fixed with 2.5% GTA in 0.1 M PB for 4 h at 4°C, followed by post-fixation with 1% osmium tetroxide (OsO_4_) in 0.1 M PB for 2 h. The specimens were dehydrated with ascending grades of alcohol and embedded in epoxy resin (Quetol 812 Mixture; Nisshin EM, Tokyo, Japan). The epoxy blocks were cut to a thickness of 60 nm. Ultrathin sections (at least five sections from each ovary) were mounted on grids and stained with uranyl acetate and lead citrate for 15 and 10 min, respectively. Transmission electron microscopy (TEM) of the stained sections was done using a model JEM-1210 transmission electron microscope (JEOL, Tokyo, Japan).

### Statistical Analyses

The results are expressed as mean ± standard error. Results were analyzed using non-parametric methods. Two groups were compared using the Mann–Whitney *U*-test (*P* < 0.05 and *P* < 0.01). The Kruskal–Wallis test was used to compare the developmental stages of three or more follicle groups with MOFs or DNOs, and multiple comparisons were performed using the Scheffé method (*P* < 0.05 and *P* < 0.01).

## Results

### Developing Follicles in the Neonatal CR Ovary

**FIGURE 1:**
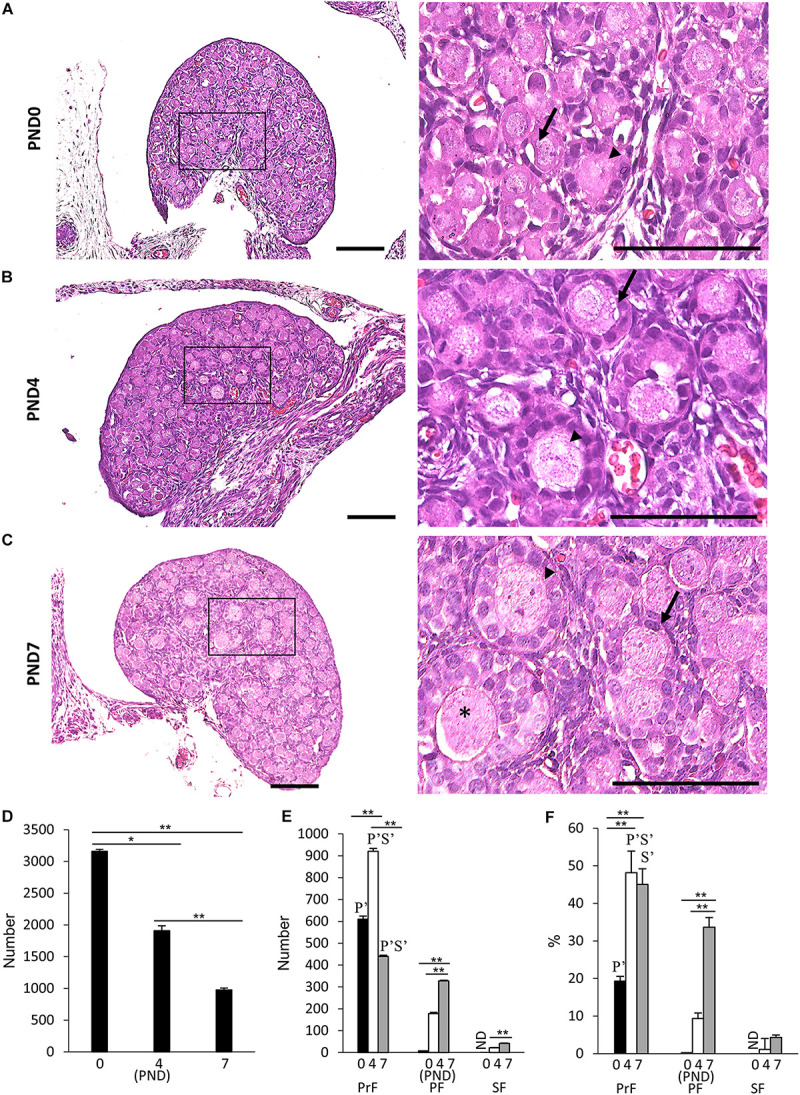
Follicular development in the neonatal ovary of cotton rats. **(A–C)** Histological observation of hematoxylin and eosin-stained sections in the ovary of neonatal cotton rats (CRs) at postnatal day (PND)0 **(A)**, PND4 **(B)**, and PND7 **(C)**. The inset figure indicates the primordial follicles (PrF) (arrow), primary follicles (PF) (arrowhead), and secondary follicles (star). The higher magnification indicates the same square area in **(A–C)**. Bars = 100 and 50 μm (inset). **(D)** The total number of developing follicles, including clusters or nests, in the neonatal CR ovary at PND0, PND4, and PND7. Values are presented as the mean + standard error. * Indicates significant difference in the total number of developmental follicles at PND0, PND4, and PND7 (Kruskal–Wallis test followed by the Scheffé method, *P < 0.05 and **P < 0.01). **(E,F)** Total number and percentage of each developing follicle classified as PrF, PF, and SF in the neonatal CR ovary at PND0, PND4, and PND7. Values indicate the mean + standard error. * Indicates significance over time within a follicle type at PND0, PND4, and PND7 (Kruskal–Wallis test followed by the Scheffé method, *P < 0.05 and **P < 0.01). Pr, P, and S denote the significance compared with PrF, PF, and SF, respectively, at the same ages (Kruskal–Wallis test followed by the Scheffé method, *P* < 0.05). Apostrophe indicate a highly significant difference (*P* < 0.01). ND, not detected. *N* = 5 for each age.

Figure 1 presents results of the histological observation of the neonatal CR ovary at PND0, PND4, and PND7. At PND0, the developing follicles were dispersed throughout the ovary as PrF and PF, with most residing at the surface (Figure 1A). At PND4, some follicles were surrounded by squamous FECs and were classified as PrF. A few of them were surrounded by a single layer of round cuboidal FECs and were classified as PF (Figure 1B). With age, CR displayed increased numbers of PrF and PF (Figures 1B,C). At PND7, the follicles had progressed to developmental stages PrF and PF, and several SFs surrounded by two or more layers of cuboidal FECs appeared in the deep cortex area (Figure 1C).

Next, we quantified the ovarian follicles at each developmental stage according to the morphology of the FEC and GC layers as mentioned in the section “Materials and Methods” and described elsewhere (Al-Mufti et al., 1988; Islam et al., 2020). Developing follicles up to SF were observed, but TF was not observed for any age. Histomorphometry revealed a significantly higher total number of oocytes at PND0 (3159.8 + 28.3), including those in nests, compared with the other ages (*P* < 0.01). Upon reaching adulthood, the ovaries of common mouse strains contain follicles of varying stages of development in the order of 3,000–5,000 (Tilly, 2003). These numbers significantly decreased with age (Figure 1D). For the number and percentage of follicles at each developmental stage, PrF showed significantly higher values (*P* < 0.01) than PF and SF at all examined ages (Figures 1E,F). For PrF, the number of follicles was significantly higher (*P* < 0.01) at PND4 than at other ages (Figure 1E). Furthermore, the number of PF and SF significantly increased (*P* < 0.01) with age (Figure 1E). The percentage of PrF was significantly higher at PND4 and PND7 than at PND0 (*P* < 0.01), and that of PF was significantly higher at PND7 than at PND0 and PND4 (*P* < 0.01), but there was no significant difference in SF.

### Development of DNOs in Neonatal CR Ovary

**FIGURE 2:**
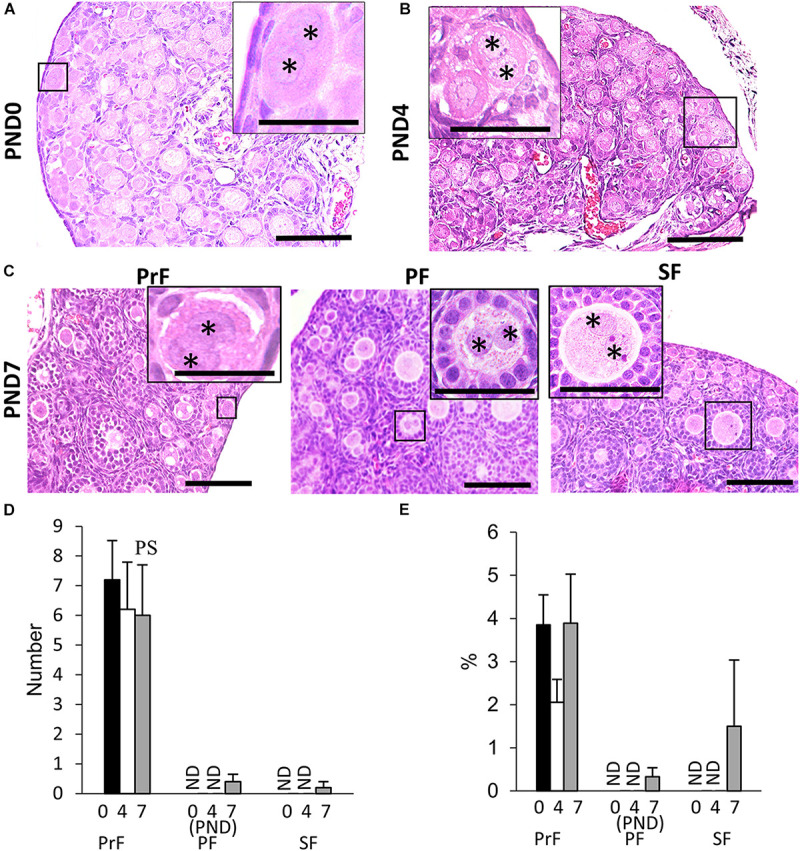
Development of double nucleated oocytes in the ovary of cotton rats. **(A–C)** Histological observation of hematoxylin and eosin-stained sections in the neonatal ovary of cotton rats (CRs) at postnatal day (PND)0 **(A)**, PND4 **(B)**, and PND7 **(C)**. Two nuclei are observed within a single oocyte follicle (SOF) classified as primordial follicle (PrF) (**A:** PND0; **B:** PND4), primary follicle (PF), and secondary follicle (SF) (**C:** PND7), respectively. Each nucleus (asterisk) shares the same cytoplasm. Bar = 100 and 50 μm (inset). **(D,E)** The total number and percentage of double nucleated oocytes (DNOs) classified as each developing follicle in neonate CR ovaries at PND0, PND4, and PND7. Values are the mean + standard error. Pr, P, and S denote the significance compared with PrF, PF, and SF, respectively, at PND7 (Kruskal–Wallis test followed by the Scheffé method, *P* < 0.05). ND, not detected. *N* = 5 at each age.

We found unique characteristics along with the developing follicles of neonatal CR ovary, DNOs (oocytes with two nuclei encased in a single follicle) at 6–8 weeks of age (Islam et al., 2020). The appearance of DNOs varies among developing follicles. At PND0 and PND4, DNOs were observed in the PrF (Figures 2A,B). With age, at PND7, we observed DNOs in PrF, PF, and SF (Figure 2C). Most of the PrF-containing DNOs appeared in the periphery of the ovary, with others distributed throughout the ovary. In histomorphology, PrF DNOs tended to be frequently observed rather than PF, and SF DNOs. The PrF DNOs might be the result of fusion of two individual oogonia during nest breaking, and PF, and SF DNOs might be the result of nuclear division of oocytes without cytoplasmic cleavage during folliculogenesis.

The histomorphometric data for DNO are shown in Figures 2D,E. Most DNOs appeared in the early developmental stages of folliculogenesis. At PND7, DNOs appeared in the PrF, PF, and SF. The percentage of DNOs in PrF at PND0 to PND7 tended to be higher than that in other developing follicles. A significant difference in the number of DNOs was observed between PrF and both PF and SF at PND7 (*P* < 0.05).

No DNOs in the ovaries of C57BL/6N mice were observed (Supplementary Figure 1).

### Development of MOFs in Neonatal CR Ovary

**FIGURE 3:**
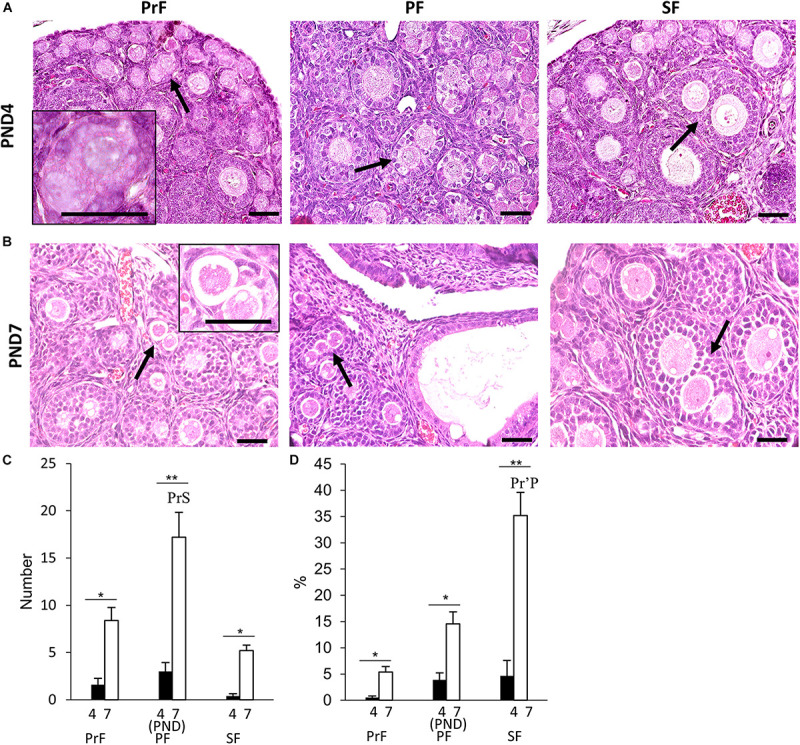
Development of multi-oocyte follicles in the neonatal ovary of cotton rats. **(A,B)** Histological observation of hematoxylin and eosin-stained sections in the neonatal ovary of cotton rats (CRs) at postnatal days (PND)4 **(A)** and PND7 **(B)**. Multi-oocyte follicles (MOFs) denoted by the arrows were observed in follicles classified as primordial follicle (PrF), primary follicle (PF), and secondary follicle (SF) **(A,B)** at PND4 and PND7, respectively. Bars = 100 and 50 μm (inset). **(C,D)** The total number and percentage of MOFs classified as each developing follicle in the neonate CR ovary at PND4 and PND7. Values the mean + standard error. * Indicates significant differences between PND4 and PND7 within follicle categories (Mann–Whitney *U*-test, *P < 0.05 and **P < 0.01). Pr, P, and S denote the significance with PrF, PF, and SF, respectively, at PND4, and PND7 (Kruskal–Wallis test followed by the Scheffé method, *P* < 0.05). Apostrophe indicate a highly significant difference (*P* < 0.01). *N* = 5 at each age.

Multi-oocyte follicles was another important unique feature of the neonatal CR ovary. At PND0, oocyte clusters and follicles dispersed throughout the ovary were evident. Their morphology was similar to that of the MOFs classified in a previous study with PrF in the CR ovaries at 6–8 weeks of age (Islam et al., 2020). Therefore, we did not consider MOFs at PND0. Subsequently, MOF, defined as a single follicle containing two or more oocytes, appeared at PND4 and PND7 in PrF, PF, and SF (Figures 3A,B), respectively (Islam et al., 2020).

For histomorphometry, MOFs classified as PF were most numerous in both PND4 and PND7 (Figure 3C). A significant difference was observed in PF MOFs compared with PrF and SF MOFs at PND7 (*P* < 0.01). SF MOFs showed significantly higher percentages than PrF MOFs at PND7 (*P* < 0.01; Figure 3D). The MOF number and percentages showed significant difference within follicle class at PND7 compared with that in PND4 (*P* < 0.01; Figures 3C,D).

**FIGURE 4:**
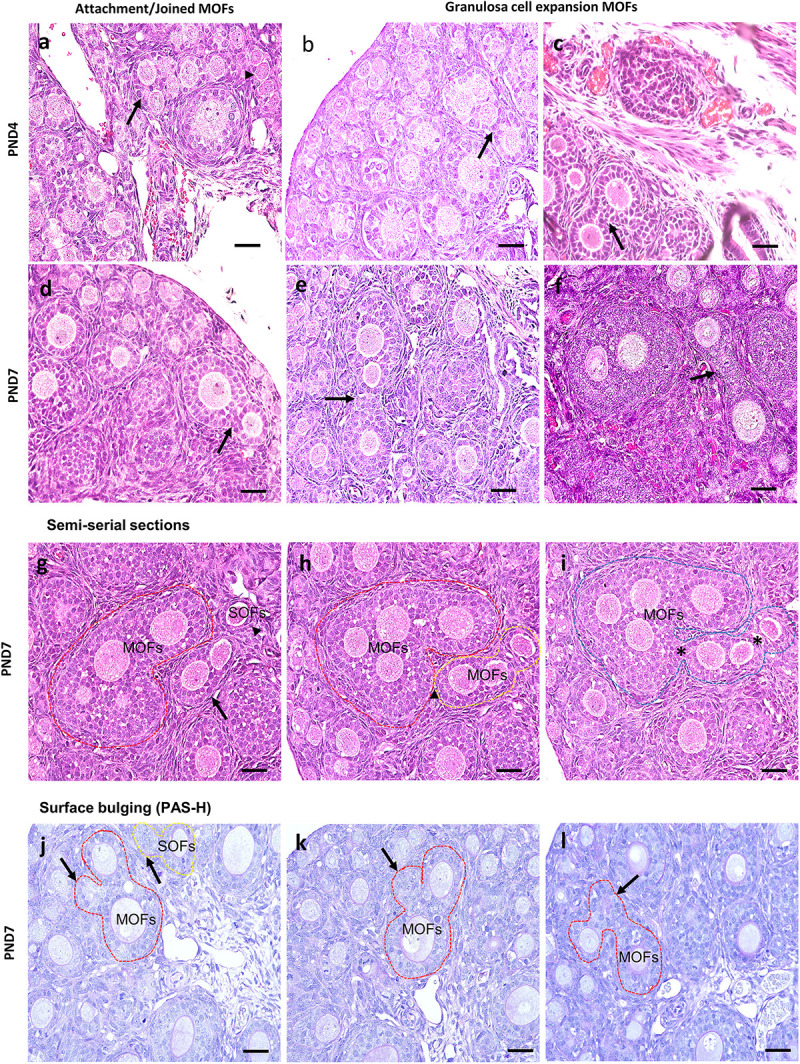
Characteristics of multi-oocyte follicles in the neonate ovary of cotton rats at postnatal day 4 and 7. **(a–f)** Histological observation of hematoxylin and eosin-stained section in the neonate ovary of cotton rats (CRs) at postnatal day (PND)4 and 7. More than three oocytes are closely attached (arrow and arrowhead) with each other to form multi-oocyte follicles (MOFs) in the single follicles classified as primary follicle (PF) (**a**; PND4), two follicles (SF and PF) joined but they are separated by granulosa cells (arrows) (**d**; PND7). Extension or invasiveness of granulosa cells (GCs) denoted by the arrows of two or more follicles in a single follicle to appear as MOFs (**b,c**: PND4; **e,f**: PND7). Scale bar = 100 μm. **(g–i)** Hematoxylin and eosin-stained semi-serial sections of ovary at PND7. Two MOFs (red circle and arrow, **g**) and one single oocyte follicle (SOF; arrowhead in **g**). The SOF is connected with nearby MOFs (yellow circle) via fine connections of granulosa cells (yellow circle, **h**) but is separated from MOFs (red circle). The MOFs are then connected via shared GCs (asterisk) between adjacent MOFs (yellow circle) to form single MOFs (blue circle in **i**). **(j–l)** Periodic acid Schiff-hematoxylin-stained section of the ovary at PND7. The outline of MOF (red circle, **j–l**) and SOF (yellow circle, **j**) shows the bulging of follicle surface (arrows in **j–l**) within the basement membrane.

Most MOFs had two oocytes in a single follicle. However, some displayed three or more oocytes in a single follicle (Figure 4). The sizes and shapes of MOFs varied compared to single oocyte follicle (SOF) as well as the developing follicles containing MOFs due to the increased number of oocytes and FECs/GCs. Moreover, morphological variation was observed in different developing follicles during folliculogenesis. Two or more oocytes were observed attached or joined to each other as PF to PF (Figure 4a: PND4), PF to SF (Figure 4d: PND7) by GCs extension. Some MOFs showed the follicle spheres linked by a bridge of GCs (Figure 4c: PND4 and 4e-f: PND7). Examination of semi-serial sections at PND7 revealed that the apparently single follicles or MOF follicles (Figure 4g) in one section were connected to those of the next sections (Figures 4h,i) by GCs. Further, some MOFs (Figures 4j–l) and SOFs (Figure 4j) at PND7 had irregular outlines showing bulging of the follicle surface within the basement membrane.

No MOFs were detected in ovaries of C57BL/6N mice (Supplementary Figure 1).

### Characterization of SOFs, MOFs, and DNOs in Neonatal CR Ovary

**FIGURE 5:**
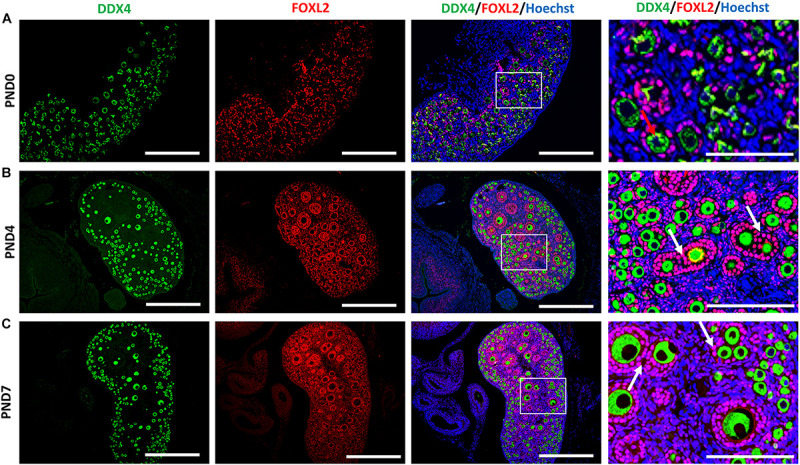
Follicular and oocyte development along with double nucleated oocytes and multi-oocyte follicles in neonate cotton rats. **(A–C)** Double immunofluorescence staining by DEAD (Asp-Glu-Ala-Asp) box polypeptide 4 (DDX4) and Forkhead box L2 (FOXL2) in ovaries of neonate cotton rats (CRs) at postnatal day (PND)0 **(A)**, PND4 **(B)**, and PND7 **(C)**. The positive signals for DDX4 (green) and FOXL2 (red) are detected in the cytoplasm of oocytes and the nuclei of follicular epithelial cells, respectively. Higher magnification (square area) of **(A–C)** indicate the development of single nucleated follicles (red arrows) and MOFs (white arrows). Nuclei are stained by Hoechst 33342 (blue). Bars = 100 and 50 μm (inset).

We examined the oocyte and follicular development stages in neonatal CR ovaries at PND0, PND4, and PND7 using double immunofluorescence of the DDX4 oocyte marker and FOXL2 FEC marker (Figures 5A–C). Immunoreactivities of DDX4 and FOXL2 were detected in the cytoplasm of oocytes and nuclei of follicular epithelial cells, respectively, throughout the observation period.

At PND0, numerous DDX4-positive oocytes were observed in the periphery of the cortices (Figure 5A). Additionally, PrF and a few PFs surrounded by squamous and cuboidal FOXL2-positive FECs were observed in the superficial and deep cortices. The PF and very few SFs surrounded by cuboidal FECs observed at the inner cortices and MOFs were first detected. They shared a common follicular area (Figure 5B) in the PND4 CR ovary. Unlike PND4, SFs with two or more layers of cuboidal GCs and MOFs were observed at PND7 (Figure 5C).

**FIGURE 6:**
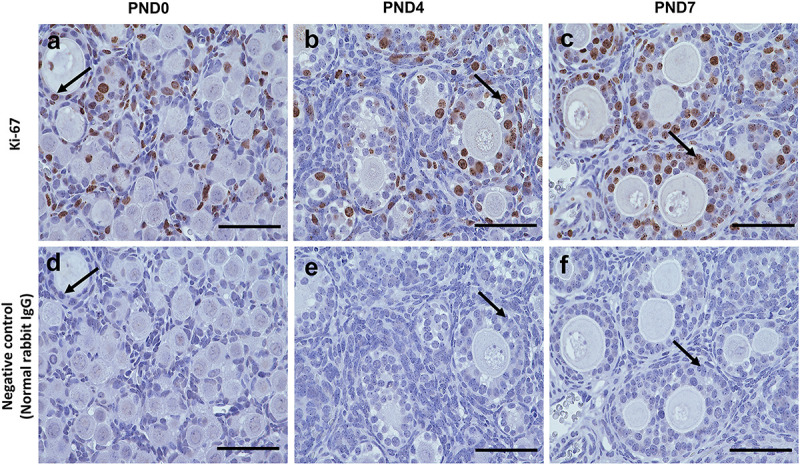
Proliferative activity of developing follicles in the neonatal ovary of cotton rats. **(a–c)** Immunohistochemistry for the assessment of proliferative activity in the developing follicles in ovaries (serial sections) of neonate cotton rats (CRs) at postnatal day (PND)0 **(a)**, PND4 **(b)**, and PND7 **(c)** by Ki-67 staining. Ki-67-positive reactions (arrows) are observed only in granulosa cells, but in single oocyte follicles (SOFs), double nucleated oocytes (DNOs), and multi-oocyte follicles (MOFs) at PND0, PND4, and PND7 (**a–c**, respectively). Panels **(d–f)** are normal rabbit IgG controls for PND0, PND4, and PND7, respectively. Scale bar = 100 μm.

However, Ki-67 staining demonstrated the proliferative activity of GCs, but not of oocytes, in the SOFs and MOFs (Figures 6a–c), and was not detectable in GCs of the ovary in normal rabbit IgG control CRs (Figures 6d–f). During the development of follicles, proliferation increased when flattened squamous GCs were converted to cuboidal GCs. As the majority of follicles were surrounded by flattened squamous GCs at PND0, we hypothesized that the formation of MOF was absent. With age, proliferation increased in the developing follicles, combined with rapid folliculogenesis, and formation of MOFs was observed at PND4. However, proliferative activity was absent in oocytes, so the formation of DNOs was unclear.

### Apoptosis in Neonatal CR Ovary Compared With Other Rodents (Mice)

**FIGURE 7:**
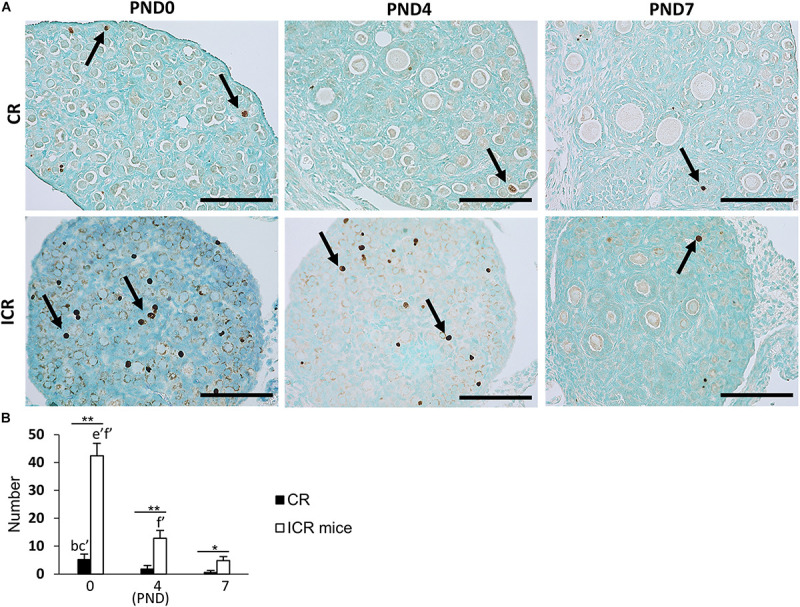
Oocyte apoptosis in neonate cotton rat compared with other rodents (mice). **(A)** Terminal deoxynucleotidyl transferase dUTP nick end labeling (TUNEL)-positive oocytes (arrows) at postnatal days (PND)0, 4, and 7 in the ovaries of neonate cotton rats (CRs) and ICR mice, respectively. **(B)** Number of TUNEL-positive apoptotic oocytes at PND0, PND4, and PND7 of CR and ICR mice, based on the analysis of five sections at each age of different ovaries. Values are the mean + standard error. Letters (a, b, c, d, e, f) denote significance with PND0, PND4, and PND7 TUNEL-positive status in CR and ICR mice, respectively (Kruskal–Wallis test followed by the Scheffé method, *P* < 0.05). *Apostrophe* beside the letters indicate a highly significant difference in ICR mice and CR at different stages (*P* < 0.01). * Indicates significant difference between PND0-CR vs. ICR, PND4-CR vs. ICR and PND7-CR vs. ICR according to the Mann–Whitney *U*-test (*P < 0.05 and **P < 0.01). *N* = five sections at each age.

We also observed the developing oocytes at PND0, PND4, and PND7 by the TUNEL assay. This experiment was done with the hypothesis that oocyte apoptosis is related to the formation of unique characteristics of the ovary in CRs. A few TUNEL-positive apoptotic oocytes in neonatal CR were observed at PND0. They were scarce in PND4 and PND7 (Figure 7A). Conversely, ICR mice had numerous TUNEL-positive apoptotic oocytes at PND0, which decreased with age (Figure 7A). The proportion of developing follicles (C57BL/6N, Supplementary Figure 1D) and apoptotic oocytes in CRs compared with that in ICRs could play a role in the formation of MOFs.

The number of TUNEL-positive apoptotic oocytes decreased with age in both CR and ICR mice, and was significantly higher in neonatal ICR mice than in neonatal CR mice (*P* < 0.01; Figure 7B). Within age, the number was also significantly higher in ICR mice compared with that in CR (*P* < 0.01).

### Ultrastructural Features of Neonatal CR Ovaries

**FIGURE 8:**
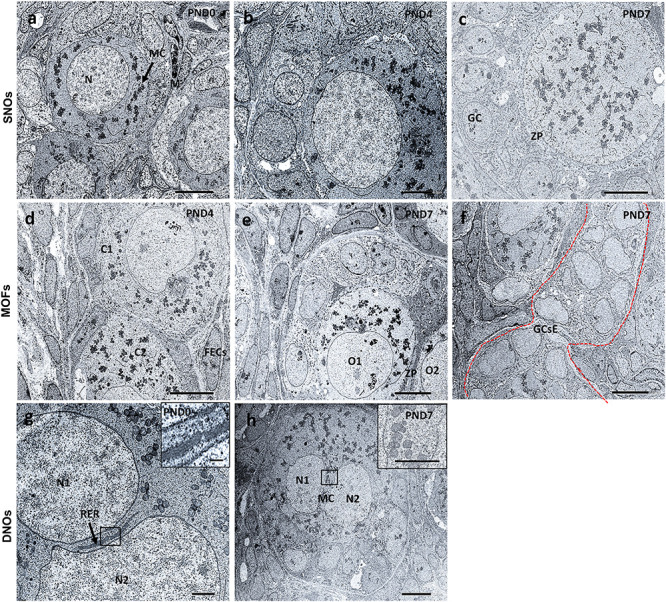
Ultrastructural features of single nucleated oocytes, multi-oocyte follicles, and double nucleated oocytes in the neonate ovary of cotton rats. Transmission electron microscopy images of the neonate ovary of cotton rats (CRs) at postnatal days (PND)0, 4, and 7. Panels **(a–c)** are the images of SNOs, where **(a)** indicates the representative primordial follicle (PrF) with a single oocyte with a clear round nucleus (N) and mitochondrial clouds (MCs, denoted by arrows) are dispersed throughout the cytoplasm of oocytes at PND0. **(b)** depicts the PF SNOs and MC aggregated to one side of the cytoplasm of follicles at PND4 and **(c)** at PND7 PF SNOs with clearly visible zona pellucida (ZP) and GCs. Panels **(d–f)** are the images of MOFs. **(d)** Depicts PrF MOFs surrounded by a common follicular epithelial cell layer and the cytoplasm (C1 and C2) are closely connected at PND4. Oocytes (O1 and O2) with a common ZP in the same cytoplasm of PF MOFs in **(e)** at PND7. With age (PND7), **(f)** shows the granulosa cell extensions, one of the developmental characteristic features of MOFs. Panels **(g,h)** are images of DNOs. **(g)** Shows DNOs (N1 and N2) that are clearly separated by rough endoplasmic reticulum (RER, denoted by arrows) and nuclei that do not have the same shape at PND0. The nuclei (N1 and N2) are in the same cytoplasm of PF DNOs separated by MC (inset) in **(h)** at PND7. Scale bar = 2 μm in **(g)**, 5 μm in **(b)**, and 10 μm in **(a,c,d,e,f)** (inset 1 μm), and **(h)** (inset 5 μm).

Results of TEM analysis of follicles and oocytes in the neonatal CR ovary are presented in Figure 8. SOF containing single nucleated oocytes (SNOs) are shown in Figures 8a–c. SOFs classified as PrF had SNO-containing mitochondrial clouds (MCs) dispersed throughout the cytoplasm that were surrounded by squamous FECs at PND0 (Figure 8a). In SOFs classified as PF, SNOs were surrounded by cuboidal FEC (or GCs) at PND4 and PND7 (Figures 8b,c). A clear zona pellucida was observed with larger PFs at PND7 (Figure 8c). These SNOs contained MCs and occasionally showed polarity in their cytoplasm at PND4 (Figure 8b).

MOF SNOs classified as PrF at PND4 (Figure 8d) and PF at PND7 (Figure 8e) were surrounded and separated by squamous and cuboidal FECs, respectively. We also determined the GC extension characteristics, which were the most important factors in the formation of MOFs at PND7 (Figure 8f).

In PrF, double nuclei were separated by rough endoplasmic reticulum (RER) at PND0 (Figure 8g). In PF, MCs were localized between double nuclei at PND7 (Figure 8h). No DNOs were detected at PND4 by TEM.

## Discussion

Ovarian development and function depend on somatic cell-oocyte communication, which is also important for follicle integrity. The breaking of germline cysts and establishment of the PrF pool for future recruitment are coordinated by the oocyte and somatic cell identity and their interactions (Matzuk et al., 2002; Tingen et al., 2009). The mechanisms by which cell–cell communication networks are established between pre-GCs and oocytes within new PrFs remain unknown. Two distinct populations of PrFs (medullary and cortical) exist in the postnatal ovary. The medullary PrFs begin to grow as soon as they are formed, whereas the cortical PrFs mature gradually over the reproductive lifespan of the animal (Hirshfield, 1992; Zheng et al., 2014). In this study, we observed neonatal CR ovaries at PND0 to PND7, where the most characteristic features of DNOs, MOFs, and fewer apoptotic oocytes were found along with the development of typical follicles. The presence of MOFs in adult ovaries has been reported in several mammals (Lucci et al., 1999; Ireland et al., 2008; Payan-Carreira and Pires, 2008). The development of neonatal CR-specific DNOs and the abundance of MOFs have been clarified as unique features during nest-breaking and folliculogenesis, compared with other rodents and mammals.

Four different hypothetical mechanisms may explain the formation of DNOs and MOFs. The first involves DNOs, via fusion of two individual oogonia during nest breaking or nuclear division of oocytes without cytoplasmic cleavage occurring during folliculogenesis. Secondly, concerning MOFs, encasement of two or more oocytes into PrF, PF, and SF may occur during follicle assembly. Thirdly, and also for MOFs, invasiveness or expansion of GCs within the follicles may occur. Finally, for MOFs, oogonia transformation to oocytes may occur or the number of apoptotic oocytes may be less. These hypotheses would be helpful to identify the potential for the formation of DNOs or MOFs in other domestic mammals, and women.

Our previous studies showed that DNOs appeared in all developing stages of the follicle, but tended to be numerous in PrF at 6–8 weeks in ovaries of CRs (Islam et al., 2020). Double nucleated cells were also observed in other tissues, such as hepatocytes or trophoblasts. These double nuclei indicated their active transcriptional activity (Donne et al., 2020). The volume of hepatocytes in human and mouse livers is approximately doubled with the doubling of DNA content, resulting from cytokinesis failure that occurs progressively during the course of postnatal development (Martin et al., 2002). DNO formation may be attributed to the fusion of two individual oogonia during nest-breaking, nuclear division without cytoplasmic cleavage during folliculogenesis, or other unknown factors. Moreover, proliferative activity was absent in the oocytes of neonatal CRs, as indicated by Ki-67 staining. There have been no reports of DNOs in other rodents or mammals, except polynucleated oocytes in humans (Dandekar et al., 1988). However, binuclear oocytes are usually found in possums (Hartman, 1926). Based on these findings, we hypothesize that DNOs in CRs form due to fusion of two individual oogonia during nest breaking or nuclear division of oocytes without cytoplasmic cleavage occurring during folliculogenesis.

In our previous study, MOFs, the characteristic feature of the CR ovary, were found in all follicle stages and more frequently in PF and SF at 6–8 weeks of age (Islam et al., 2020). Similarly, we found MOFs in neonates at PND4 and PND7. PND0 was excluded because follicles that were clustered or dispersed throughout the ovary appeared to be MOFs. The presence of MOFs in adult ovaries has been reported in mammals (Lucci et al., 1999; Ireland et al., 2008; Payan-Carreira and Pires, 2008). The present data show that the number and percentages of MOFs increased from PND4 to PND7. This observation strongly supports the hypothesis that the vast majority of MOFs found in peripubertal CRs are generated by connection or fusion of adjacent growing follicles. Oogonia can proliferate in fetal mammals, and most of develop into oocytes by the initiation of the first meiotic division. Oogonia divide synchronously with intercellular bridges due to incomplete cytokinesis (Pepling et al., 2007). The bridges generally disappear in oocytes. Therefore, dysregulated cell division during oocytogenesis may also contribute to the formation of MOFs. It has been proposed that MOFs may be derived from the failure of oocyte nest breakdown during the early stages of folliculogenesis (Zeilmaker et al., 1983; Safran et al., 1998; Lucci et al., 1999; Oktem and Urman, 2010; Oliveira et al., 2017). However, in CRs at PND4, nest-breaking appeared to be almost complete, implying the separation of the nest to each follicle dispersed throughout the ovary. Therefore, the dysregulation of this process might also contribute to the production of MOFs in CRs at PND4 and PND7.

Another factor may be the encasement of multiple oocytes in PrF during folliculogenesis due to their more rapid developmental rate compared to the differentiation of the surrounding somatic cells (Telfer and Gosden, 1987; Kennedy, 1924; Lucci et al., 1999; Balciuniene et al., 2006). Furthermore, the presence of irregularly shaped follicles with bulges and projections of the follicle surface (Islam et al., 2020), follicles with breaches of the follicle wall with apparent migration of GC into the ovarian stroma, and linked follicles sharing GCs is suggestive of follicle joining (Figures 4g–l). Moreover, irregular outlines of MOF showed the bulging of the follicle surface within the basement membrane (Figures 4j–l). Oocytes, GCs, and theca cells form follicle units within the ovary with basement membrane separating the granulosa and theca cells compartments. Signaling between all three of these cell types is essential to support follicle development, oocyte development, and ovulation (Christensen et al., 2017). The FOXL2-positive FECs showed that the projection of cells started at PND4. These observations strongly suggest a sequence of events responsible for follicle fusion. According to such morphologic indices, it can be hypothesized that the formation of bulges on the surface of some growing follicles and the eventual breaching of the follicle wall, which allows GCs to invade the perifollicular stroma, constitute the first steps for MOF generation. However, although the proliferative activity of MOFs has been described in mammals (Oliveira et al., 2017), Ki-67 staining presently demonstrated proliferative activity of GCs, but not of oocytes, in the MOFs of CRs. A recent study suggested the invasive capacity of GCs, which could contribute to the connection of the follicles and the generation of the MOFs in the peripubertal rat ovary (Gaytán et al., 2014). Based on these considerations, it is likely that MOFs form either by fusion or GC extension/invasiveness during the development of follicles.

Morphologically, apoptosis is found in ovarian follicles throughout fetal and adult life (Arends et al., 1990). Apoptosis is an essential component of ovarian function and development. However, in neonatal CR, the number of TUNEL-positive apoptotic oocytes was comparatively lower than that in other rodents (e.g., ICR mice). The proportion of developing follicles (C57BL/6N, Supplementary Figure 1D) and apoptotic oocytes in CRs compared to that in ICR could play a role in the formation of MOFs. Cells with a high regeneration and division rate, and cells under endocrine control are particularly susceptible to apoptosis (Wyllie, 1987). Therefore, germ cells, especially oogonia, are prone to be involved in apoptosis (Qi et al., 2008). Germ cell proliferation and apoptosis during the period of development leads to primordial follicle formation (Fulton et al., 2005), but a lower number of apoptotic cells in the ovary may stimulate the formation of MOFs.

The thickness of the zona pellucida in mammals is usually similar among oocytes (Payan-Carreira and Pires, 2008), but differs in MOFs in 6–8-week-old CR ovaries (Islam et al., 2020). MCs tended to localize throughout the cytoplasm of SOFs, MOFs, and DNOs. However, we found a difference in the orientation of MCs between SOFs and MOFs in CR ovaries at 6–8 weeks of age. Those of MOFs seemed to be localized to the cytoplasm, including long distance areas (Islam et al., 2020). These have been termed Balbiani bodies (Bbs). Bbs contain large RNA-protein granules and are universally conserved in the oocytes of insects (Jaglarz et al., 2003), fish (Marlow and Mullins, 2008), rodents (Pepling et al., 2007), and humans (Albamonte et al., 2013). Bbs are transient structures, as they only exist in the dormant oocytes and are dispersed once the oocyte is activated (Pepling et al., 2007). Although the functional significance of Bbs in CRs is unclear, the polarity of Bbs in *Xenopus* oocytes is associated with germinal granules responsible for the determination of germ cell fate (Kloc et al., 2008). Light microscopy revealed nuclear polarity in MOF oocytes. Ultrastructurally, the polarity of MOF oocytes appeared to be caused by the aggregation of mitochondria in the cytoplasm.

The fertility status of MOF is not well known. Some authors have suggested that MOFs may ovulate (Bysted et al., 2001; Reynaud et al., 2009). Other authors have suggested that the smallest of the oocytes in a MOFs might be arrested in development and will probably degenerate, so that all might disappear except one or that all might undergo atresia and never reach the stage of extrusion of the first polar body (Kennedy, 1924). As fertility status of MOF is not well clarified, the abundance of MOFs in CRs could be used as a model for studying such disturbances in other domestic mammals, and women.

**FIGURE 9:**
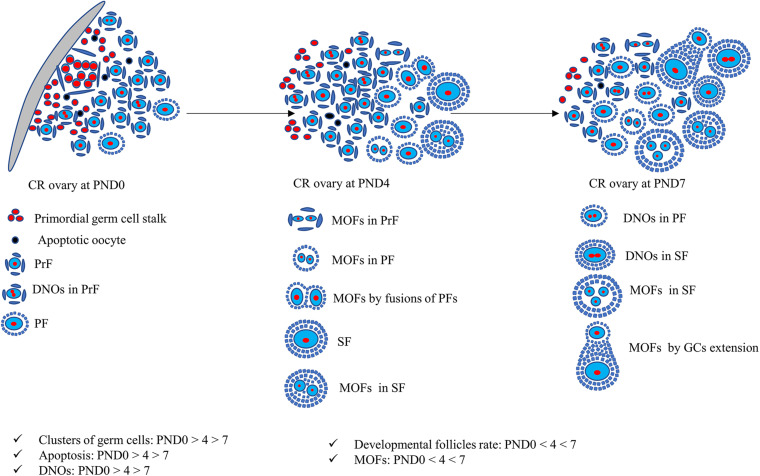
Schematic presentation of development of unique characteristics along with developing follicles in neonatal cotton rat ovaries. At postnatal day (PND)0, follicles are dispersed throughout the ovary with few apoptotic oocytes. The follicles appear as clusters and develop into primordial follicles (PrFs). A few are primary follicles (PF) surrounded by single layer squamous and cuboidal follicular epithelial cells (FECs), respectively. With age, at PND4, follicles progress to develop into secondary follicles (SF). One of the unique characteristics of PrF is double nucleated oocytes (DNOs). Moreover, at PND4, besides DNOs in PrF, another important characteristic of multi-oocyte follicles (MOFs) is the formation in PrF, PF, and SF. At PND7, DNOs, and MOFs are formed in all developing stages of follicles on SF.

The findings of our study of neonatal CR ovaries is summarized in Figure 9. Clusters of primordial germ cells and apoptosis both decrease, but the proportion of MOFs increases with age. The follicles develop up to the secondary stage, with the formation of DNOs and MOFs. A lower number of apoptotic cells indicates rapid folliculogenesis. The rate of MOFs increases with age, resulting in the mechanism of fusion or GC extension. On the other hand, DNOs decrease, but are observed in developing follicles with age, due to nuclear division without any cytoplasmic cleavage.

Thus, follicular development and the formation of unique phenotypes implicate CR as a useful model to study folliculogenesis and oocytogenesis as well as abnormalities in humans and other animals. Further studies focusing on the ovulation- and fertility-related features of oocytes derived from DNOs and MOFs are required to clarify the characteristics of the reproductive function of DNOs and MOFs.

## Data Availability Statement

The original contributions presented in the study are included in the article/Supplementary Material, further inquiries can be directed to the corresponding author/s.

## Ethics Statement

The animal study was reviewed and approved by Hokkaido Institute of Public Health (approval no. K27-03) and The Faculty of Veterinary Medicine, Hokkaido University (approval no. 20-0012).

## Author Contributions

MI, OI, TN, TI, MM, YE, and YK conceived and designed the experiments. MI, OI, TN, MM, TC, and TI performed the sampling of cotton rat ovaries. MI, OI, TN, MM, and YO performed the experiments. MI, OI, and YK analyzed the data and wrote the manuscript. All the authors contributed to the article and approved the submitted version.

## Conflict of Interest

The authors declare that the research was conducted in the absence of any commercial or financial relationships that could be construed as a potential conflict of interest.
