# Supplementary figures and images for: Developmental Changes of the Ovary in Neonatal Cotton Rat (Sigmodon hispidus)
Source: Front Physiol. 2021 Jan 13;11:601927. doi: 10.3389/fphys.2020.601927 (PMC7838641; doi:10.3389/fphys.2020.601927)

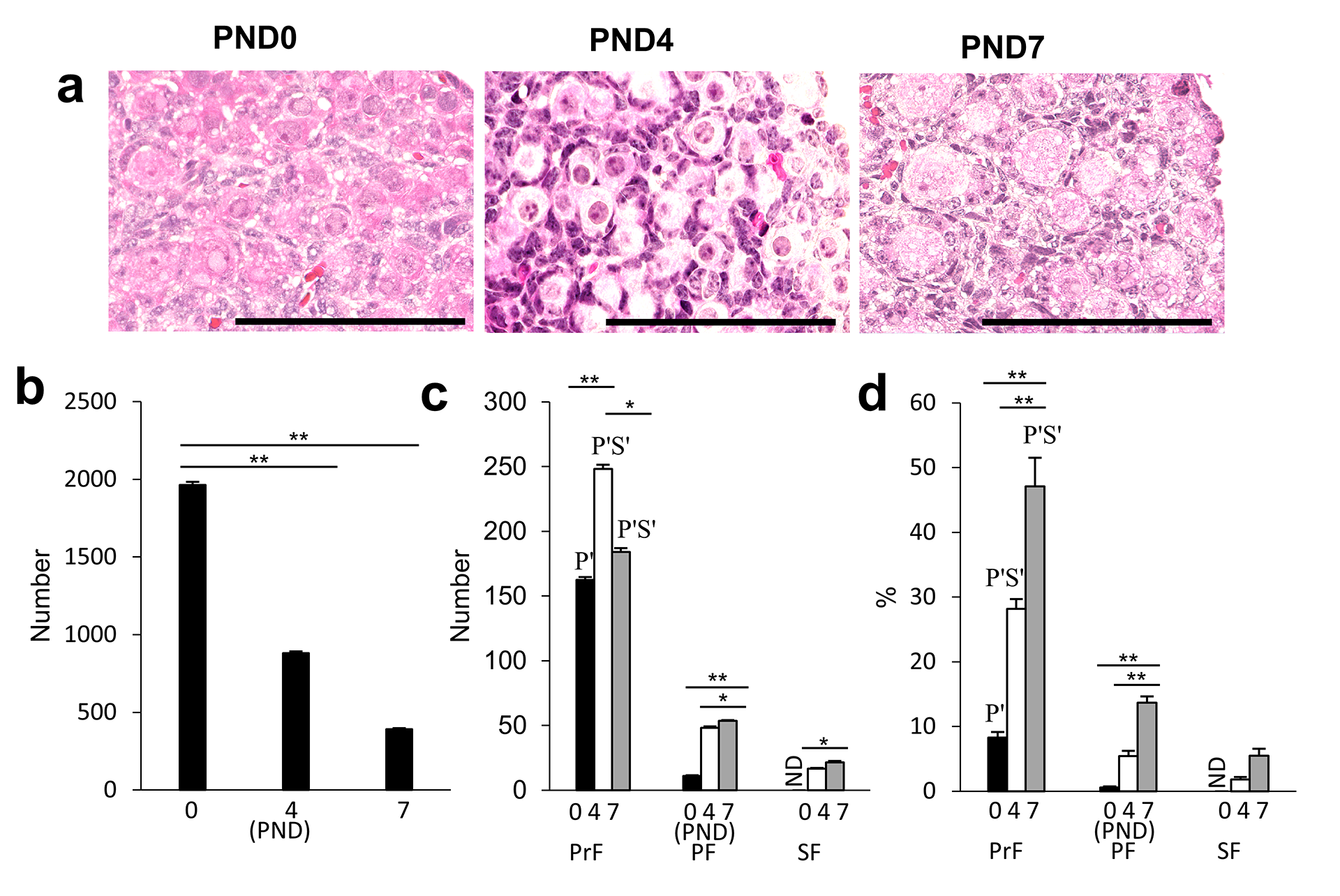

Supplement: Supplementary file 1 [file Image_1.TIF]
