# Supplementary material for: Developmental Changes of the Ovary in Neonatal Cotton Rat (Sigmodon hispidus)
Source: Front Physiol. 2021 Jan 13;11:601927. doi: 10.3389/fphys.2020.601927 (PMC7838641; doi:10.3389/fphys.2020.601927)
Supplement: Supplementary file 2 [file Table_1.docx]

**Supplemental Fig. 1. Follicular appearance in the neonatal ovary of C57BL/6N mice**

(**a**) Histological observation of hematoxylin and eosin-stained sections in the ovaries of neonatal C57BL/6N mice at PND0, PND4, and PND7. Bars = 50 µm. (**b**) The total number of developing follicles, including nests, in ovaries of neonatal C57BL/6N mice at PND0, PND4, and PND7. Values are presented as the mean + standard error. * indicates significant difference in the total number of developmental follicles at PND0, PND4, and PND7 (Kruskal-Wallis test followed by the Scheffé method, **P* < 0.05 and ***P* < 0.01). (**c–d**) The total number and percentage of each developing follicle classified as PrF, PF, and SF in ovaries of neonatal C57BL/6N mice at PND0, PND4, and PND7. Values are the mean + standard error. * indicates significance over time within a follicle type at PND0, PND4, and PND7 (Kruskal-Wallis test followed by the Scheffé method, **P* < 0.05 and ***P* < 0.01). Pr, P, and S denote the significance compared with PrF, PF, and SF, respectively, at the same ages (Kruskal-Wallis test followed by the Scheffé method, *P* < 0.05). Apostrophe indicate a highly significant difference (*P* < 0.01). ND: not detected. N = 5 at each age.
